# Antimicrobial Activity of Quinoline-Based Hydroxyimidazolium Hybrids

**DOI:** 10.3390/antibiotics8040239

**Published:** 2019-11-28

**Authors:** Daniel Insuasty, Oscar Vidal, Anthony Bernal, Edgar Marquez, Juan Guzman, Braulio Insuasty, Jairo Quiroga, Laura Svetaz, Susana Zacchino, Gloria Puerto, Rodrigo Abonia

**Affiliations:** 1 Departamento de Química y Biología, Universidad del Norte, Km 5 vía Puerto Colombia, Barranquilla 081007, Colombia; oorjuela@uninorte.edu.co (O.V.); awbernal@uninorte.edu.co (A.B.); ebrazon@uninorte.edu.co (E.M.); 2 Institute for Insect Biotechnology, Justus-Liebig-University of Giessen, 35392 Giessen, Germany; Juan.D.Guzman-Vasquez@agrar.uni-giessen.de; 3 Research Group of Heterocyclic Compounds, Department of Chemistry, Universidad del Valle, A. A. Cali 25360, Colombia; braulio.insuasty@correounivalle.edu.co (B.I.); jairo.quiroga@correounivalle.edu.co (J.Q.); 4 Área Farmacognosia, Facultad de Ciencias Bioquímicas y Farmacéuticas, Universidad Nacional de Rosario, Suipacha 531, Rosario 2000, Argentina; laurasvetaz@hotmail.com (L.S.); szaabgil@citynet.net.ar (S.Z.); 5 Laboratorio de Micobacterias, Instituto Nacional de Salud, Bogotá 111321, Colombia; gpuerto@ins.gov.co

**Keywords:** quinoline-based hydroxyimidazolium hybrids, antimicrobial activity, antifungal activity, tuberculosis, cytotoxicity

## Abstract

Eight quinoline-based hydroxyimidazolium hybrids **7a–h** were prepared and evaluated in vitro against a panel of clinically important fungal and bacterial pathogens, including mycobacteria. Hybrid compounds **7c–d** showed remarkable antifungal activity against *Cryptococcus neoformans* with a minimum inhibitory concentration (MIC) value of 15.6 µg/mL. Against other opportunistic fungi such as *Candida* spp. and *Aspergillus* spp., these hybrids showed MIC values of 62.5 µg/mL. Regarding their antibacterial activity, all the synthetic hybrids demonstrated little inhibition of Gram-negative bacteria (MIC ≥50 µg/mL), however, hybrid **7b** displayed >50% inhibition against *Klebsiella pneumoniae* at 20 µg/mL and full inhibition at 50 µg/mL. Moreover, this hybrid was shown to be a potent anti-staphylococcal molecule, with a MIC value of 2 µg/mL (5 µM). In addition, hybrid **7h** also demonstrated inhibition of *Staphylococcus aureus* at 20 µg/mL (47 µM). Hybrids **7a** and **7b** were the most potent against *Mycobacterium tuberculosis* H37Rv with MIC values of 20 and 10 µg/mL (46 and 24 µM), respectively. The **7b** hybrid demonstrated high selectivity in killing *S. aureus* and *M. tuberculosis* H37Rv in comparison with mammalian cells (SI >20), and thus it can be considered a hit molecule for mechanism of action studies and the exploration of related chemical space.

## 1. Introduction

The intensive use of antibacterial and antifungal drugs has led to an increase in difficult-to-eradicate infections [1]. In recent years, the trend in reducing infectious disease mortality has been threatened by the emergence of strains of bacteria that are no longer susceptible to the currently available antimicrobial agents such as the Gram-negative *Acinetobacter baumannii*, *Pseudomonas aeruginosa*, or the Gram-positive *Enterococcus faecium*, or *Staphylococcus aureus* added to the multi- and extensively- drug-resistant *Mycobacterium tuberculosis*. Regarding fungi, they are also a source of concern in antifungal chemotherapy because many fungi can be opportunistic pathogens seriously affecting immunocompromised patients, and some of them, such as *Candida albicans*, *Cryptococcus neoformans,* and *Aspergillus* spp. can cause complicated systemic infections that are associated with high mortality rates [2].

With the rise of the difficult-to-eradicate infectious diseases, the need for new antimicrobial agents is urgently needed. A promising strategy for the development of new antimicrobial drugs is the synthesis of molecular hybrids containing two or more covalently joined antimicrobial pharmacophores within a single molecule [3,4,5].

**Figure 1:**
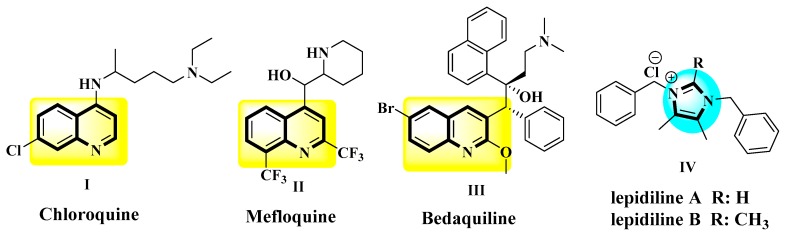
Some examples of biologically important quinoline- and imidazolium-derivatives.

Quinoline moiety is historically important because it is present in the *Cinchona* alkaloids quinine and quinidine, which were the first useful treatment for malaria. Based on the activity of these natural products, several quinoline-based molecules have shown to be effective inhibitors of essential proteins from microbial pathogens [6]. For that, synthetic antimalarials have been developed, and some of them, such as amodiaquine, chloroquine (**I** in Figure 1), mefloquine (**II** in Figure 1), and piperaquine are still used clinically today, as they are recommended by the WHO [7]. One of the by-products of the synthesis of chloroquine was identified as an active antibacterial principle in 1960, and further research led to the discovery of nalidixic acid in 1962 and later to the fluoroquinolone class of antibacterials [8]. Their clinical importance is evident, as these were highly active against most Enterobacteriaceae, which includes common pathogens such as *Escherichia coli*, *Salmonella* spp., *Yersinia pestis*, and others, and in addition these molecules are unique to targeting bacterial DNA topoisomerases [9]. Moreover, other quinoline-containing compounds have been developed as antibacterial drugs, and one remarkable example is bedaquiline (**III** in Figure 1). This diarylquinoline derivative is a first-in-class anti-tuberculosis drug, acting by inhibition of mycobacterial ATP synthase and approved to treat multiple-drug resistant tuberculosis [10].

Although quaternary ammonium salts such as benzalkonium and cetylpyridinium chlorides salts have been used for a long time as antiseptics in a variety of pharmaceutical and cosmetic products [11,12], the imidazolium salts are not used as antiseptics, nor are they constitutive fragment of any drug thus far. In contrast, the imidazole ring is a common moiety in antifungal chemotherapeutic agents such as clotrimazole, ketoconazole, and miconazole [13,14,15]. Interestingly, the imidazolium moiety is present in the lepidilines (**IV** in Figure 1), which are in fact antitumoral natural products from the Neotropical plant *Lepidium meyenii* [16]. Synthetic natural products analogs showed potent antimicrobial [17] and antitumoral activities [18,19]. The interesting biological properties of imidazolium salts may be explained by the possibility of not only engaging in ion-dipole and hydrogen bond interactions but also to participate in acid-base reactions and to coordinate metal atoms. This versatility opens the door to tuning their selectivity by covalent linking with appropriate steric and electrostatic scaffolds directed to particular biochemical targets.

In this work, eight quinoline-based hydroxyimidazolium hybrids **7a–h** were prepared and evaluated against a panel of clinically important fungal and bacterial pathogens, including *Mycobacterium tuberculosis* H37Rv.

## 2. Results and Discussion

### 2.1. Chemistry

**Figure 2:**
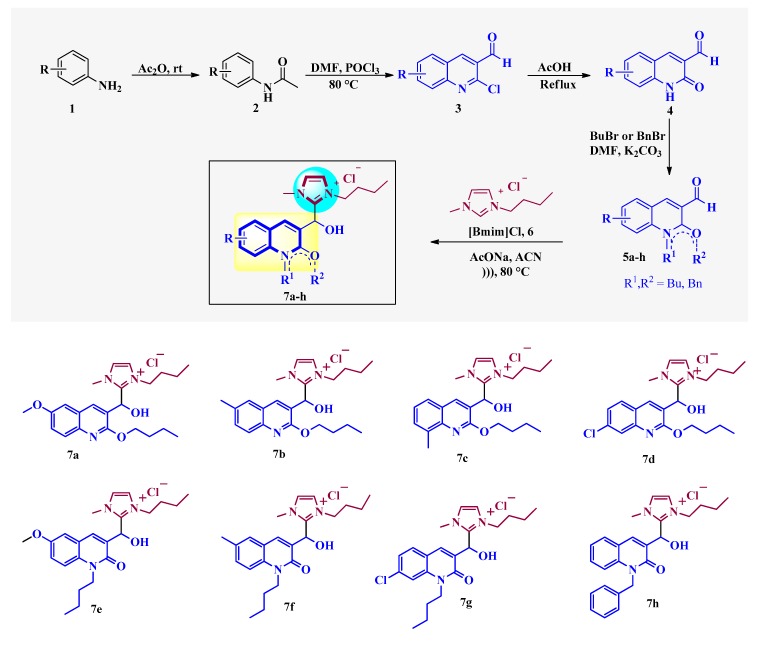
Synthesis of quinoline-based hydroxyimidazolium hybrids **7a–h**.

The synthesis of the quinoline-based hydroxyimidazolium hybrids **7** was carried out by following our previously reported methodology [20]. Briefly, the non-commercial 3-formyl-2-oxo-quinoline and 3-formyl-2-alkoxy-quinoline precursors **5a–h** were synthesized by a Meth-Cohn reaction [21], followed by acid hydrolysis and alkylation (Figure 2). Subsequently, the aldehydes **5a–h** were subjected to reaction with 3-butyl-1-methylimidazolium chloride ([Bmim]Cl) **6** in the presence of AcONa and ACN as a solvent, under ultrasound irradiation at 80 °C during 1–7 h. This straightforward procedure afforded the corresponding quinoline-based hydroxyimidazolium hybrids **7a–h** in 60–91% yield. Structures of hybrids **7** were confirmed by ^1^H NMR (see Figure S1 in Supplementary Materials).

### 2.2. Antifungal Activity

Hybrids **7a–h** were tested in vitro for antifungal activity against the most common causes of invasive fungal diseases *Candida albicans* and *Cryptococcus neoformans* and the molds *Aspergillus niger*, *Aspergillus fumigatus*, and *Aspergillus flavus*. The minimum inhibitory concentration (MIC) of all hybrids was determined with the clinical and laboratory standards institute (CLSI) microbroth dilution methods M27-A3 and M38-A2 [22,23]. Hybrids with MICs >250 µg/mL were considered inactive; in the range of 250 to 62.5 µg/mL, with moderate or low activity, and hybrids with MICs ≤31.25 µg/mL were considered high activity.

Results in Table 1 clearly show that *C. neoformans* were the most susceptible sp., with all hybrids showing some degree of antifungal activity with MIC values between 15.6 and 250 µg/mL. Instead, MIC values of **7a–h** against *C. albicans* were in the range of 62.5 to (>250 µg/mL) with two hybrids (**7e** and **7h**) showing to be inactive (MIC >250 µg/mL). Regarding *Aspergillus* spp., they were the less sensitive spp, since 5 hybrids (**7a**, **7b**, **7e–g**) (of the 8 hybrids tested) were inactive.

**Figure 3:**
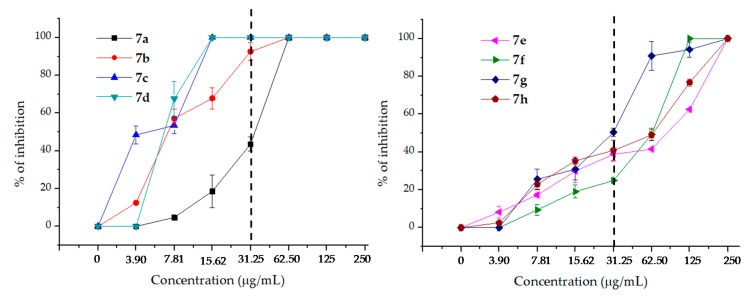
Dose–response curves of hybrids **7a–d** (left) y **7e–h** (right) against *C. neoformans* ATCC 32264.

In order to have a deeper look at the antifungal behavior of hybrids **7** against *C. neoformans*, the percentages of inhibition of *C. neoformans* for each hybrid **7a–h** at concentrations from 250 to 3.90 µg/mL (obtained by two-fold dilutions) were determined. Results are recorded in Table 2 and represented in Figure 3, where the differences in the activity of the 7 hybrids **7** against *C. neoformans* can be clearly observed.

Table 2 shows that the hybrids derived from 2-alkoxy-quinolines (**7a–d**) were more potent than those from 2-oxo-quinolines (**7e–h**), and this can clearly be seen in Figure 3.

The dotted lines show the percentages of inhibition of the hybrids at 31.25 µg/mL and is useful to clearly observe the behavior of the hybrids at lower concentrations that are considered highly active. In Figure 3, it can be corroborated that **7a–d** possess higher anti-cryptococcal activity than **7e–h** since the inhibition percentages of **7a–d** at concentrations ≤31.25 µg/mL are clearly higher than those of **7e–h.**

From the above results, some preliminary structure–activity relationships can be drawn: (i) The methoxy group in position C-6 (hybrid **7a**), induced a relative lower activity than the methyl group in the same position (hybrid **7b**), as shown in Table 1 and Table 2. (ii) The methyl group in position C-8 of the hybrid **7c** seemed to confer a significant increase in antifungal potency compared with the methyl group in position C-6 of hybrid **7b**. (iii) Hybrids **7c** and **7d** showed the same MIC (15.62 µg/mL) values, although **7c** seemed to be more potent than **7d**, as evidenced by the much stronger inhibition displayed by **7c** at 3.9 µg/mL, (see Table 2). This finding could suggest that either the chlorine atom at position C-7 in **7d** decreased the activity or the methyl group in position C-8 (hybrid **7c**) increased the antifungal effect, or that both played a significant role in the activity. (iv) The same detrimental trend by the chlorine atom in position C-7 on the antifungal activity was also observed when comparing the *N*-butylated hybrids **7f** (6-Me) (MIC = 125 mg/L) with **7g** (7-Cl) (MIC = 250 mg/L. (v) Nevertheless, the methyl group in position C-6 (hybrid **7b**) induced a lower activity even than the chlorine atom at position C-7 in **7d**, affording MIC values of 15.62 and 31.25, respectively, Table 2. (vi) Additionally, comparison of the activity of the *O*-butylated hybrid **7b** (MIC = 31.25) with the *N*-butylated hybrid **7f** (MIC = 62.5), both possessing a 6-Me substituent, indicated that not only this substituent and its position contributed to the activity of the synthesized hybrids **7**.

### 2.3. Antibacterial Activity

**Figure 4:**
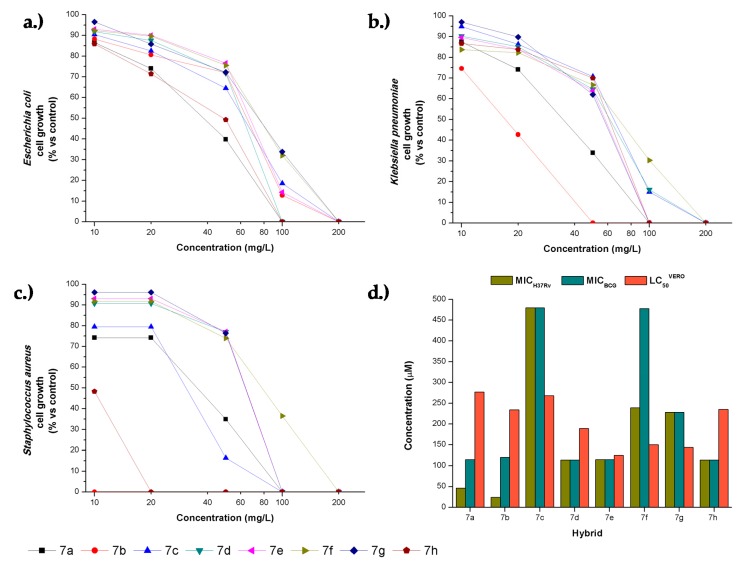
Antibacterial activity of hybrids **7a–h**. Comparative dose–response curves of hybrids **7a–h** against (**a**) *E. coli*, (**b**) *K. pneumoniae,* and (**c**) *S. aureus*. (**d**) Comparison of the concentrations required for anti-mycobacterial and cytotoxic effects against *M. tuberculosis* H37Rv, *M. bovis* BCG, and (Verda Reno) VERO cell line.

The hybrids **7a–h** were tested against two Gram-negative microorganisms; *Escherichia coli* and *Klebsiella pneumoniae*, a Gram-positive bacteria, *Staphylococcus aureus,* and two acid-fast slow-growing mycobacteria *Mycobacterium tuberculosis* H37Rv and *Mycobacterium bovis* BCG. Results for each hybrid were expressed as minimum inhibitory concentration values (MICs, µg/mL and µM), as shown in Table 3. Chloramphenicol was used as a positive control for *E. coli*, *K. pneumoniae*, and *S. aureus*, and isoniazid for mycobacteria. All the synthetic hybrids showed little activity against the Gram-negative organisms with MIC values ≥50 µg/mL. Against *K. pneumoniae*, hybrids **7a** and **7b** showed to be the most potent, as evidenced by their moderate growth inhibition at high concentration (Figure 4). The hybrid **7b** demonstrated a potent anti-staphylococcal activity with a MIC value of 2 µg/mL (5 µM). The hybrid **7h** also demonstrated a significant inhibition against *S. aureus* with a MIC value of 20 µg/mL (47 µM) and should also be considered as a hit molecule for exploring related chemical space. Hybrids **7a** and **7b** showed significant inhibition against *M. tuberculosis* H37Rv with MIC values of 20 and 10 µg/mL (46 and 24 µM), respectively. The remaining hybrids **7d**, **7e,** and **7h** displayed moderate growth inhibition against the virulent H37Rv strain with MIC values of 50 µg/mL (–115 µM). A similar trend of activity was found against *M. bovis* BCG, confirming their antimycobacterial effect.

The anti-mycobacterial response of the hybrids towards the non-virulent *M. bovis* BCG and the virulent *M. tuberculosis* H37Rv was compared (Table 3). A correlation in MIC between the two organisms was found to be 0.8 for this dataset. Substitution at C-6 as in **7a** and **7b** seemed to be essential for increasing the anti-mycobacterial effect, whereas benzyl substitution on the nitrogen atom, as in **7h**, seemed to promote anti-staphylococcal activity. A number of antimicrobial quinolone hybrids have been published in recent literature [24,25], and although some of these hybrids were active against Gram-negative bacteria, only the hybrids with chloroquine and triclosan [26], showed inhibition of *S. aureus* at concentrations comparable (MIC–2 mg/L) to our best results. Against *Mycobacterium tuberculosis*, the hybrids of quinolone and pyrazole displayed inhibitory values (MIC 12.5–50 mg/L) similar to our results [27]. However, evidently other quinoline derivatives are more potent antimycobacterial agents [28] and were developed, for instance, as the currently approved drug bedaquiline.

In summary, the hybrid **7b** represents a promising molecule showing significant antibacterial effect against *S. aureus* (5 µM) and *M. tuberculosis* H37R (24 µM), for which the study of its mechanism of action should be undertaken to assess its potential development as an anti-infective agent. In this direction, some previous reports indicated that quinoline hybrids, along with other nitrogen-containing heterocycles have shown to bind to heme and hemozoin unities, but also DNA, acetylcholinesterase, or to affect prostaglandin production [29,30,31,32,33]. The hits discovered in this study open the door for the exploration of a focused library of compounds and to deepen the study of their antimicrobial mechanism of action.

### 2.4. Cytotoxicity and Selective Index

The cytotoxic activity of the hybrids **7a–h** was evaluated by an MTT (3-(4,5-dimethyl-2-thiazolyl)-2,5-diphenyl-2*H*-tetrazolium bromide) assay on VERO Cells (ATCC^®^ CCL-81™) with peroxide as a positive control and dimethyl sulfoxide (DMSO) as a negative control. VERO cell line growth was expressed as half-lethal concentration (LC_50_) values and are summarized in Table 4.

Examination of cytotoxic activity of the hybrids **7a–h** on the green monkey VERO cell line showed, in general, a low effect with LC_50_ values higher than 125 µM (Table 4). The 2-alkoxy-quinolines (**7a–d**) showed to be slightly less cytotoxic (LC_50_ ~ 189–277 µM) than the 2-oxo-quinolines (**7e–h**) (LC_50_ ~ 125–235 µM). The selectivity index (SI) was calculated as the ratio between LC_50_ on VERO cells and MIC against bacteria. As the cytotoxic concentrations were relatively similar, the hybrids with lower MIC values such as **7a**, **7b**, and **7h**, showed higher selectivity. Specifically, the hybrid **7b** showed high SI values of 117 and 23.4 for *S. aureus* and *M. tuberculosis* H37Rv. The SI is an indirect measure of the therapeutic window, and it can serve as a predictor of safety during in vivo trials for a given bacterial infection.

## 3. Materials and Methods

### 3.1. Antifungal Activity

#### 3.1.1. Microorganisms and Media

For the antifungal evaluation, standardized strains from the American type culture collection (ATCC, Manassas, VA, USA), and CEREMIC (CCC, Centro de Referencia en Micología, Facultad de Ciencias Bioquímicas y Farmacéuticas, Rosario, Argentina) were used: *C. albicans* ATCC 10231, *C. neoformans* ATCC 32264, *A. flavus* ATCC 9170, *A. fumigatus* ATTC 26934, *A. niger* ATCC 9029. Strains were grown on Sabouraud-chloramphenicol agar slants for 48h at 30 °C, maintained on slopes of Sabouraud-dextrose agar (SDA, Oxoid, Cambridge, UK) and sub-cultured every 15 days to prevent pleomorphic transformations. Inocula were obtained according to reported procedures [22,23] and adjusted to 1–5 × 10^3^ cells with colony-forming units (CFU)/mL.

#### 3.1.2. Antifungal Susceptibility Testing

Minimum inhibitory concentration (MIC) of each hybrid was determined by using broth microdilution techniques according to the guidelines of the Clinical and Laboratory Standards Institute for yeasts (M27-A3) [22] and for filamentous fungi (M38 A2) [23]. MIC values were determined in RPMI-1640 (Sigma-Aldrich, St Louis, MO, USA) buffered to pH 7.0 with MOPS (3-(*N*-morpholino)propanesulfonic acid). Microtiter trays were incubated at 35 °C for yeasts and *Aspergillus* spp. MICs were visually recorded at 48h for yeasts, and at a time according to the control fungus growth, for *Aspergillus* spp. For the assay, stock solutions of the hybrids were 2-fold diluted with RPMI-1640 from 250 to 3.90 µg/mL (final volume = 100 µL) and a final DMSO concentration ≤1%. A volume of 100 µL of inoculum suspension was added to each well, with the exception of the sterility control where sterile water was added to the well instead. Amphotericin B (Sigma-Aldrich, St Louis, MO, USA) was used as positive control. Endpoints were defined as the lowest concentration of drug resulting in total inhibition (MIC) of visual growth compared to the growth in the control wells containing no antifungal drug.

#### 3.1.3. Fungal Growth Inhibition Percentage Determination

This determination was performed with the yeast *C. neoformans* ATCC 32264. For the assay, compound test wells (CTWs) were prepared with stock solutions of each hybrid in DMSO (maximum concentration ≤1%), diluted with RPMI-1640, to final concentrations of 250–3.90 μg/mL. An inoculum suspension (100 μL) was added to each well (final volume in the well = 200 μL). A growth control well (GCW) (containing medium, inoculum, and the same amount of DMSO used in a CTW, but compound-free) and a sterility control well (SCW) (sample, medium, and sterile water instead of inoculum) were included for each fungus tested. Microtiter trays were incubated in a moist, dark chamber at 30 °C for 48h for both yeasts. Microplates were read in a Versa Max microplate reader (Molecular Devices, Sunnyvale, CA, USA). Amphotericin B was used as positive control. Tests were performed in triplicate. Reduction of growth for each compound concentration was calculated as follows: % of inhibition = 100−(OD _405_ CTW−OD_405_ SCW)/(OD_405_ GCW−OD_405_ SCW).

### 3.2. Antituberculosis and Antibacterial Activity

The agar dilution spot culture growth inhibition (SPOTi) assay [34,35] was performed to evaluate the minimum inhibitory concentration (MIC) values of the synthetic hybrids against the laboratory strain *M. tuberculosis* H37Rv in a biosafety level 3 laboratory of the National Health Institute in Bogota. A stock solution of the hybrids was prepared in DMSO at a concentration of 200 mg/mL. Dilutions of the compounds were prepared in DMSO in 24 well plates, 2 mL of each dilution was dispensed in each well at 200, 100, 50, 20, and 10 mg/mL. The final volume was 2 mL of molten Middlebrook 7H10 medium (HiMedia, Mumbai, India) supplemented with 0.5% glycerol and 10% oleic acid, albumin, dextrose, and catalase (OADC, BD, USA) were added to the wells. An inoculum having a cell density of 10^6^ CFU/mL was prepared from a 2-week culture of *M. tuberculosis* H37Rv (ATCC 27294) grown in Löwenstein–Jensen medium slants at 37 °C. Two microliters of the diluted inoculum were dispensed in the middle of the agar from each well, and the plates were incubated for 2–3 weeks at 37 °C. Isoniazid was included as a positive control at 10, 1, 0.1, 0.05, and 0.01 μg/mL concentrations. After the incubation period, the plates were observed, and the MIC was determined as the minimum concentration on which growth was not observed. The experiment was repeated on a different day observing exactly the same results. The SPOTi agar dilution method was also employed for MIC determination against *M. bovis* BCG. The cells were passaged first in Middlebrook 7H9 and then spotted into Middlebrook 7H10 medium with supplement albumin, 0.5% glycerol, dextrose, and sodium chloride (NaCl). The plates were incubated for 1 week at 37 °C. The concentrations tested were 200, 100, 50, 20 and 10 μg/mL and isoniazid was used as a positive control.

The evaluation of the hybrids against a panel of two Gram-negative microorganisms *E. coli* (see Table S1 in Supplementary Materials) and *K. pneumoniae* (see Table S2 in Supplementary Materials), and the Gram-positive bacteria *S. aureus* (see Table S3 in Supplementary Materials), with a varied pattern of drug-susceptibility was performed on LB broth by two-fold serial dilution with a maximum concentration of the synthetic hybrids at 200, 100, 50, 20, and 10 μg/mL. Growth was determined by optical density measurements at 640 nm every 120 min for 36 h. Chloramphenicol was used as a positive control, and the experiments were performed in duplicate.

### 3.3. Cytotoxicity on VERO Cell Line

The VERO cell line (ATCC^®^ CCL-81™) was cultured in Dulbecco Modified Eagle Medium (DMEM) supplemented with 10% bovine fetal serum (FBS) and 1% streptomycin-penicillin and passaged twice before the assay in 21 cm^2^ cell culture Petri dishes at 37 °C in 5% CO_2_ incubator and 100% humidity. The cells were then cultured in 96 well plates for 24h before the assay to a cell density of 90.000 cells per well. A 10 mg/mL hybrid stock solution was prepared in DMSO, following 2-fold serial dilution until 0.01 mg/mL. Finally, 5 μL were transferred to each plate containing the VERO cells and incubated for 48h. Hydrogen peroxide was used as a positive control and DMSO as a negative control under the same dilution conditions. After 24h of incubation, culture media was changed for 100 μL of DMEM media without FBS freshly prepared with MTT solution at 5 mg/mL, and the plates were further incubated for 2h. The media was then removed, and 100 μL of DMSO was added to each well. After 30 min incubation, the absorbance was read at 540 nm on a microplate reader (FLUOstar Omega) [36]. The experiment was performed in duplicate on different days with different cell cultures and different stock of the hybrids. The IC_50_ values were determined by interpolation from the mean absorbance data of 100% viability (negative control) and 0% viability (positive control).

## 4. Conclusions

In summary, we report the antimicrobial activity of eight quinoline-based hydroxyimidazolium hybrids **7a–h** against panels of fungal and bacterial pathogens, including mycobacteria. Hybrid compounds **7c–d** showed the highest antifungal activity against *C.* with MIC values of 15.6 µg/mL each one. Furthermore, all hybrids showed promising antibacterial activity, although, compound **7b** presented the strongest activity against *S. aureus* and *M. tuberculosis H37Rv* with MIC values of 2 µg/mL (5 µM) and 10 µg/mL (24 µM), respectively.

## Figures and Tables

**Table 1:** Minimum inhibitory concentration (MIC in µg/mL) of hybrids **7a–h**.

Structure	Hybrid	R_1_	R_2_	R_3_	R_4_	*Ca*	*Cn*	*Afu*	*Afl*	*Ani*
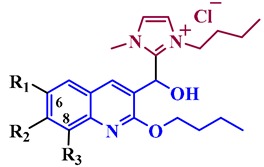	**7a**	OMe	H	H	-	125	62.5	i	i	i
	**7b**	Me	H	H	-	62.5	62.5	i	i	i
	**7c**	H	H	Me	-	62.5	15.6	62.5	62.5	62.5
	**7d**	H	Cl	H	-	62.5	15.6	62.5	62.5	62.5
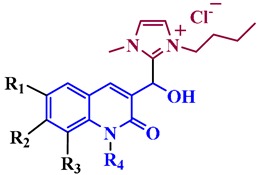	**7e**	OMe	H	H	Bu	i	250	i	i	i
	**7f**	Me	H	H	Bu	250	125	i	i	i
	**7g**	H	Cl	H	Bu	250	62.5	i	i	i
	**7h**	H	H	H	Bn	i	250	250	250	250
**AmpB**						0.78	0.25	0.50	0.50	0.50

Antifungal activity was determined with the microbroth dilution assay following the clinical laboratory standards institute (CLSI) guidelines; inactive (i) = MIC >250 µg/mL; Ca: *Candida albicans* ATCC10231, Cn: *Cryptococcus neoformans* ATCC32264, An: *Aspergillus niger* ATCC9029, Afl: *Aspergillus flavus* ATCC9170, Afu: *Aspergillus fumigatus* ATCC26934. AmpB: *Amphotericin* B; Bu = Butyl; Bn: benzyl.

**Table 2:** Percentages of inhibition of *C. neoformans* ATCC 32264 by hybrids **7a–h.**

Comp.	Concentration in µg/mL							MIC in µg/mL
	250	125	62.50	31.25	15.62	7.81	3.90	
**7a**	100	100	100	43.6 ± 3.86	18.60 ± 8.59	4.72 ± 1.39	0	62.5
**7b**	100	100	100	92.58 ± 4.81	67.84 ± 5.66	57.14 ± 4.93	12.54 ± 0.36	31.25
**7c**	100	100	100	100	100	53.45 ± 4.42	48.41 ± 4.63	15.62
**7d**	100	100	100	100	100	67.59 ± 9.19	0	15.62
**7e**	100	62.46 ± 0.30	41.46 ± 0.58	38.79 ± 3.79	29.92 ± 6.13	17.19 ± 0.91	8.23 ± 3.18	250
**7f**	100	100	49.28 ± 3.20	24.91 ± 0.15	19.01 ± 3.40	9.34 ± 2.90	0	62.5
**7g**	100	94.15 ± 4.15	90.90 ± 7.60	50.44 ± 2.09	30.94 ± 5.61	25.50 ± 5.35	0	250
**7h**	100	76.77 ± 1.91	48.99 ± 3.00	46.84 ±5.32	35.32 ± 2.02	22.76 ± 2.87	2.52 ± 0.56	250
**AmpB**	100	100	100	100	100	100	100	0.25

**Table 3:** Minimum inhibitory concentration values against *E. coli, K. pneumoniae, S. aureus, M. tuberculosis* H37Rv and *M. bovis* BCG for the synthetic hybrids **7a–h**.

Hybrid	MICs in µg/mL (µM)				
	*E. coli*	*K. pneumoniae*	*S. aureus*	*M. bovis* BCG	*M. tuberculosis* H37Rv
**7a**	100 (231)	100 (231)	100 (231)	50 (115)	20 (46)
**7b**	200 (480)	50 (120)	2 (5)	50 (120)	10 (24)
**7c**	200 (480)	200 (480)	100 (240)	200 (480)	200 (480)
**7d**	100 (229)	200 (457)	100 (229)	50 (114)	50 (114)
**7e**	200 (462)	100 (231)	100 (231)	50 (115)	50 (115)
**7f**	200 (478)	200 (478)	200 (478)	200 (478)	100 (239)
**7g**	200 (228)	100 (114)	100 (114)	100 (228)	100 (228)
**7h**	100 (228)	100 (228)	20 (47)	50 (114)	50 (114)
**chloramphenicol**	20 (62)	20 (62)	20 (62)	nd	nd
**isoniazid**	nd	nd	nd	0.05 (0.36)	0.05 (0.36)

nd = not determined.

**Table 4:** Cytotoxic activity (LC_50_) in VERO cells and selectivity index (SI) of the active synthetic hybrids **7a–h**, for *E. coli, K. pneumoniae, S. aureus, M. tuberculosis H37Rv and M. bovis BCG*.

Hybrid	Selectivity Index (SI) (SI = LC_50_/MIC)					
	*Cytotoxicity LC_50_-Vero cells*	*E. coli*	*K. pneumoniae*	*S. aureus*	*M. bovis* BCG	*M. tuberculosis* H37Rv
**7a**	277 ± 14.6	2.77	2.77	2.77	5.54	13.9
**7b**	234 ± 4	1.17	4.68	117	4.68	23.4
**7c**	268 ± 5.69	1.34	1.34	2.68	1.34	1.34
**7d**	189 ± 14.5	1.89	0.94	1.89	3.78	3.78
**7e**	125 ± 22	0.62	1.25	1.25	2.5	2.50
**7f**	150 ± 12.2	0.75	0.75	0.75	0.75	1.50
**7g**	144 ± 8.9	1.44	1.44	1.44	1.44	1.44
**7h**	235 ± 6.5	2.35	2.35	11.8	4.70	4.70

## Supplementary Materials

The following are available online at https://www.mdpi.com/2079-6382/8/4/239/s1, Table S1. Percentages of inhibition of *Escherichia coli* by hybrids **7a–h**, Table S2. Percentages of inhibition of *Klebsiella pneumoniae* by hybrids **7a–h**, Table S3. Percentages of inhibition of *Staphylococcus aureus* by hybrids **7a–h**. Figure S1. Copies of ^1^H NMR spectra for compounds **7a–h**.
