# Supplementary material for: Antimicrobial Activity of Quinoline-Based Hydroxyimidazolium Hybrids
Source: Antibiotics (Basel). 2019 Nov 28;8(4):239. doi: 10.3390/antibiotics8040239 (PMC6963836; doi:10.3390/antibiotics8040239)

Supplementary Materials

For

Antimicrobial activity of quinoline-based hydroxyimidazolium hybrids

Daniel Insuasty[1*], Oscar Vidal[1], Anthony Bernal[1], Edgar Marquez[1], Juan Guzman[2], Braulio Insuasty[3], Jairo Quiroga[3], Laura Svetaz[4], Susana Zacchino[4], Gloria Puerto[5] and Rodrigo Abonia[3*]

[1] Departamento de Química y Biología, Universidad del Norte, Km 5 vía Puerto Colombia, Barranquilla 081007, Colombia; insuastyd@uninorte.edu.co (D.I.), oorjuela@uninorte.edu.co (O.V.), awbernal@uninorte.edu.co (A.B.), ebrazon@uninorte.edu.co (E.M.)
[2] Institute for Insect Biotechnology, Justus-Liebig-University of Giessen, 35392 Giessen, Germany; Juan.D.Guzman-Vasquez@agrar.uni-giessen.de (J.G.)
[3] Research Group of Heterocyclic Compounds, Department of Chemistry, Universidad del Valle, A. A. 25360 Cali, Colombia; braulio.insuasty@correounivalle.edu.co (B.I.), jairo.quiroga@correounivalle.edu.co (J.Q.), rodrigo.abonia@correounivalle.edu.co (R.A.)
[4] Área Farmacognosia, Facultad de Ciencias Bioquímicas y Farmacéuticas, Universidad Nacional de Rosario, Suipacha 531, 2000 Rosario, Argentina; szaabgil@citynet.net.ar (S.Z.)
[5] Laboratorio de Micobacterias, Instituto Nacional de Salud, Bogotá, 111321, Colombia; gpuerto@ins.gov.co (G.P.)

Table of Contents

Tables of percentages of inhibition for antibacterial activity (S2)

Copies of ^{1}H NMR spectra for compounds **7a-h** (S3-S6)

Tables of percentages of inhibition for antibacterial activity

Table S1. Percentages of inhibition of *Escherichia coli* by hybrids **7a-h**

Escherichia coli								
**Concentrations in µg/mL**	**Hybrids**							
	**7a**	**7b**	**7c**	**7d**	**7e**	**7f**	**7g**	**7h**
**10**	13.4 ± 0.02	11.7 ± 0.06	9.6 ± 0.01	8.2 ± 0.05	7 ± 0.02	7.7 ± 0.03	3.5 ± 0.07	14.1
**20**	26 ± 0.02	19.4 ± 0.09	17.5 ± 0.01	12.4 ± 0.04	10 ± 0.006	10.4 ± 0.01	14.2 ± 0.05	28.6
**50**	60.2 ± 0.02	28 ± 0.05	35.5 ± 0.02	28.2 ± 0.01	23.4 ± 0.01	24.5 ± 0.02	27.8 ± 0.02	50.7
**100**	100	87.2 ± 0.05	81.5 ± 0.03	100	85.7 ± 0.04	68 ± 0.06	66.2 ± 0.001	100
**200**	100	100	100	100	100	100	100	100

Table S2. Percentages of inhibition of *Klebsiella pneumoniae* by hybrids **7a-h**

Klebsiella pneumoniae								
**Concentrations in µg/mL**	**Hybrids**							
	**7a**	**7b**	**7c**	**7d**	**7e**	**7f**	**7g**	**7h**
**10**	12.5 ± 0.05	25.4 ± 0.01	5.1± 0.03	9.8 ± 0.07	10.4 ± 0.03	16.3 ± 0.01	3.0 ± 0.02	13.3 ± 0.01
**20**	25.9 ± 0.02	57.3 ± 0.03	13.7 ± 0.01	14.9 ± 0.05	16.2 ± 0.01	17.8 ± 0.01	10.2 ± 0.02	16.2 ±0.03
**50**	66.1 ± 0.003	100	29.3 ± 0.01	35.4 ± 0.02	36.2 ± 0.003	33.3 ± 0.007	38.0 ± 0.01	30.0 ± 0.01
**100**	100	100	85,0 ± 0.01	83.9 ± 0.03	100	69.7 ± 0.08	100	100
**200**	100	100	100	100	100	100	100	100

Table S3. Percentages of inhibition of *Staphylococcus aureus* by hybrids **7a-h**

Staphylococcus aureus								
**Concentrations in µg/mL**	**hybrids**							
	**7a**	**7b**	**7c**	**7d**	**7e**	**7f**	**7g**	**7h**
**10**	13.2 ± 0.04	100	13.8 ± 0.1	5.8 ± 0.04	5.2 ± 0.05	7.5 ± 0.03	4.4 ± 0.06	100
**20**	25.8 ± 0.02	100	20.5 ± 0.05	9.3 ± 0.04	6.9 ± 0.05	8.2 ± 0.03	3.90 ± 0.03	100
**50**	65.1 ± 0.03	100	83.6 ± 0.03	23 ± 0.02	22.9 ± 0. 1	26 ± 0.3	23.6 ± 0.05	100
**100**	100	100	100	100	100	63.5 ± 0.01	100	100
**200**	100	100	100	100	100	100	100	100

Figure S1. Copies of ^{1}H NMR spectra for compounds 7a-h

Compound 7a

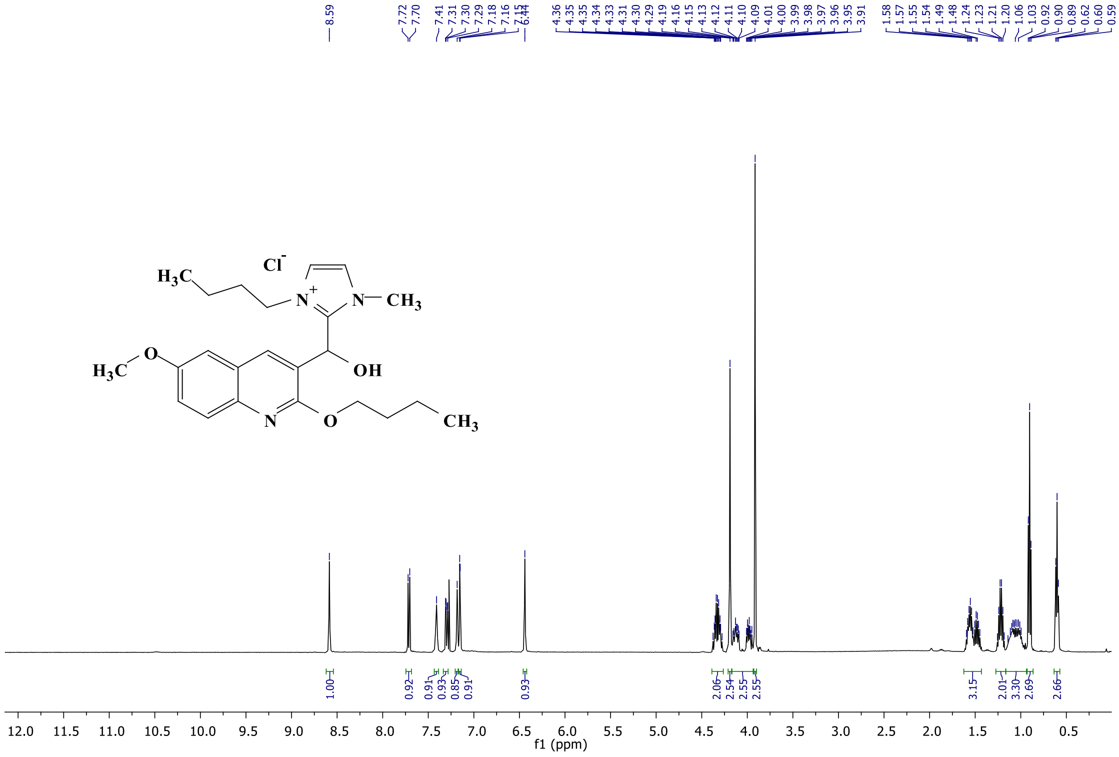

Compound 7b

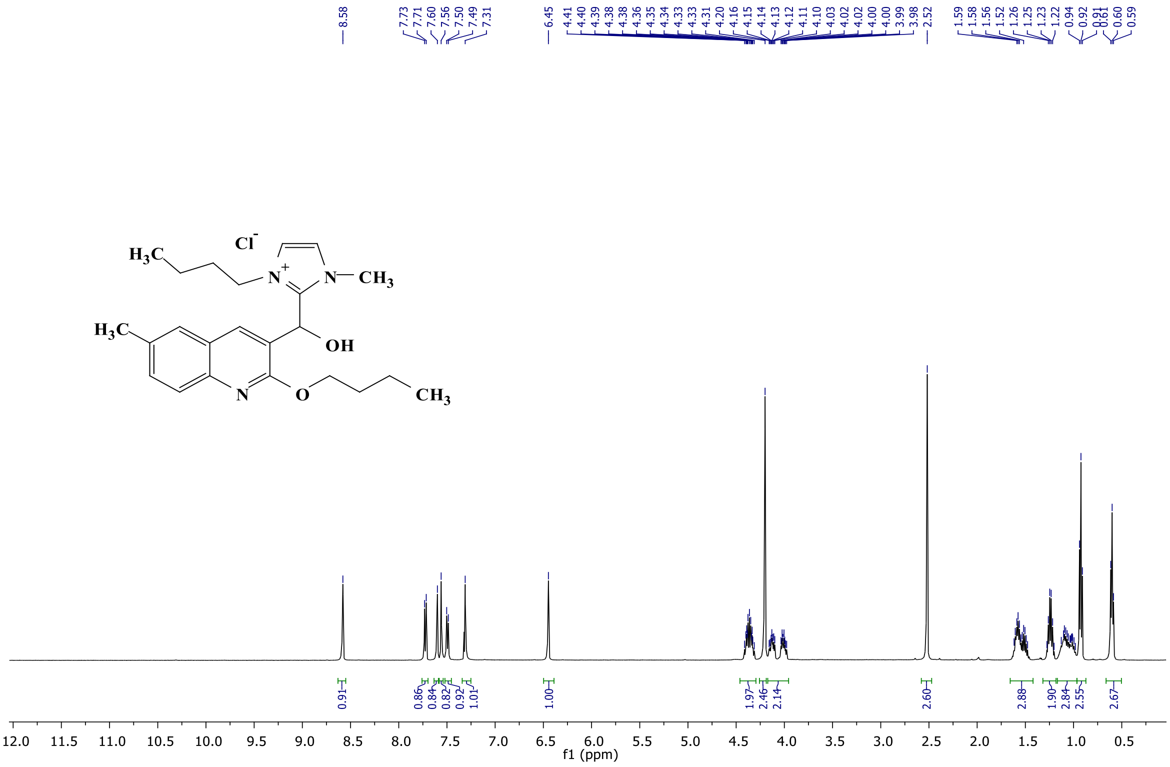

Compound 7c

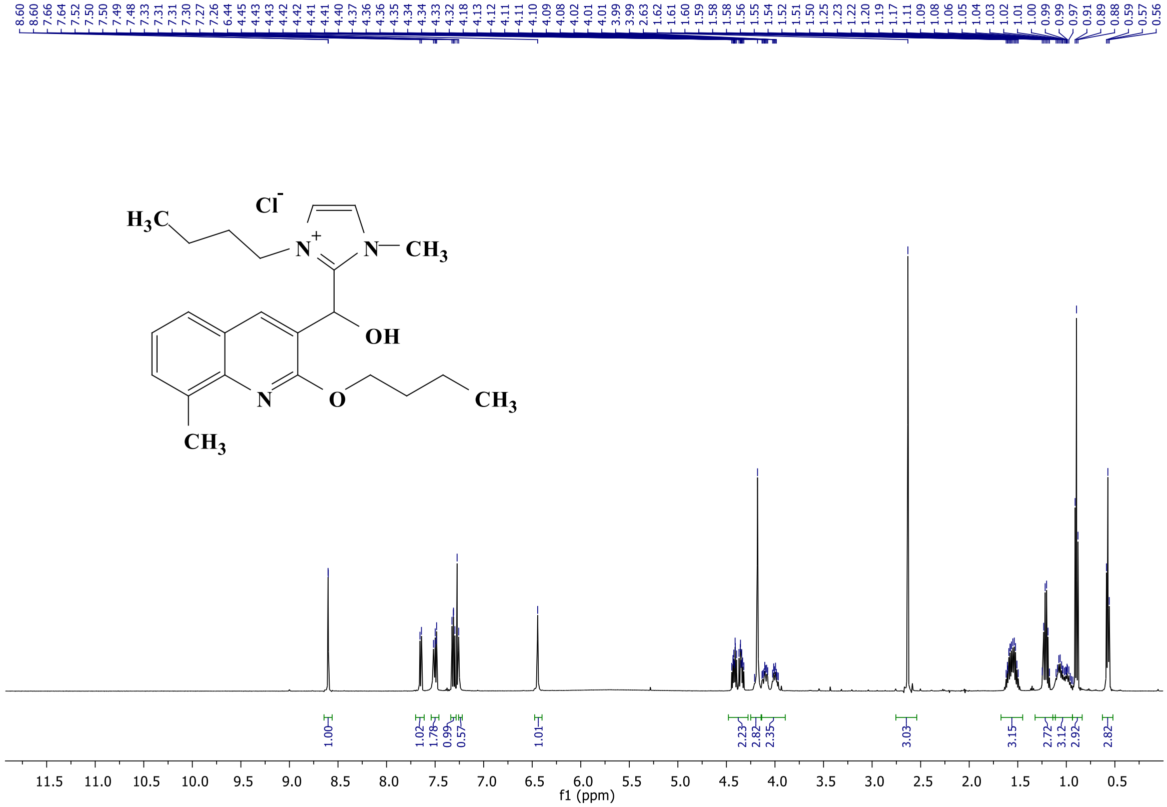

Compound 7d

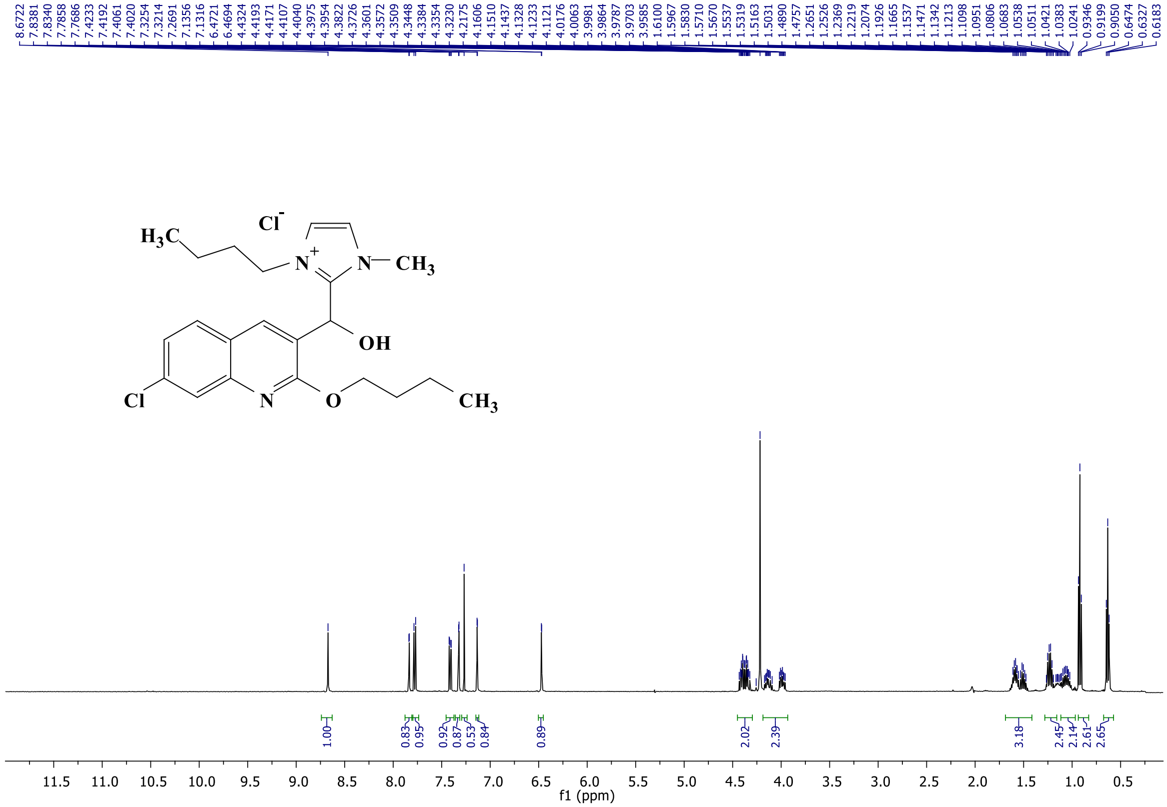

Compound 7e

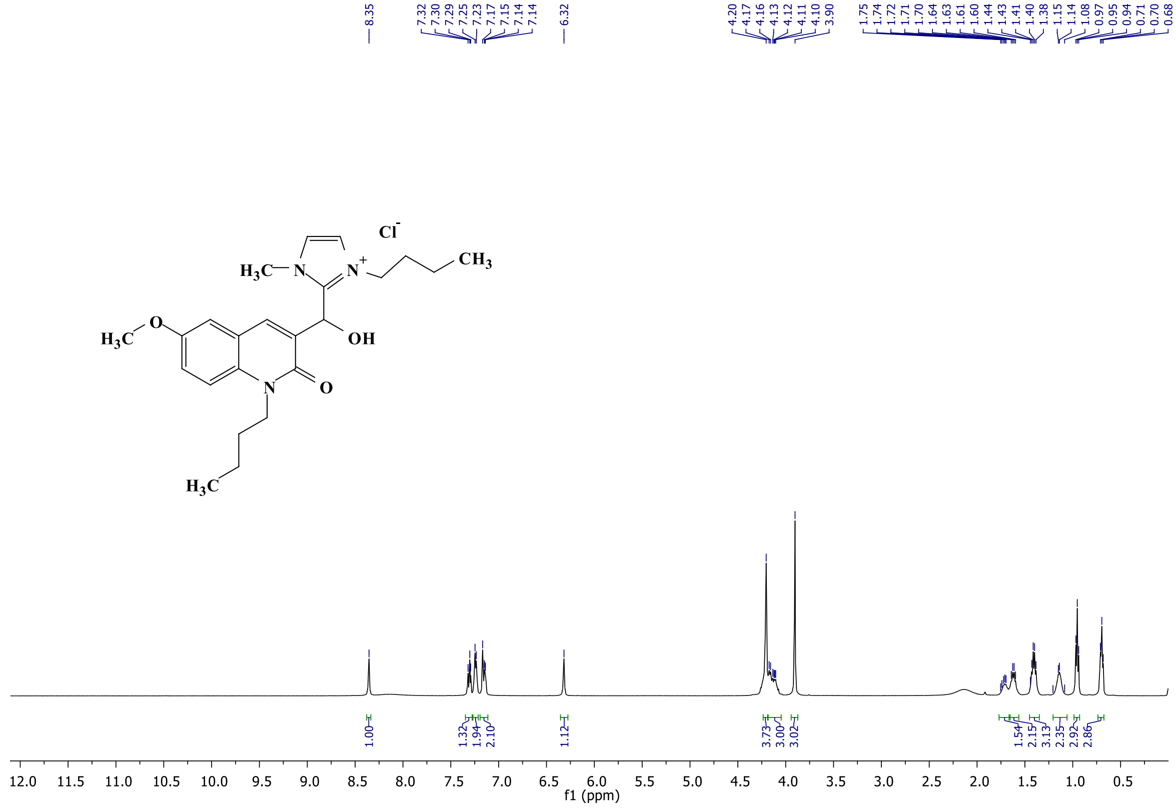

Compound 7f

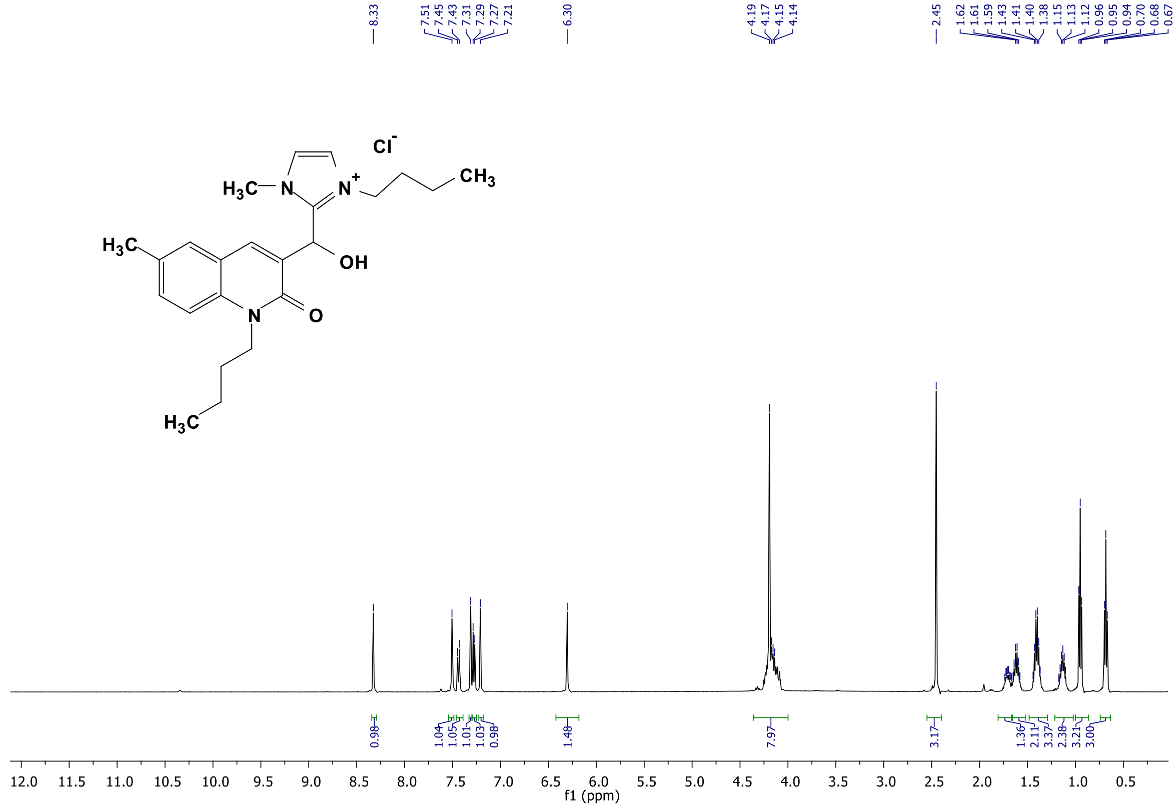

Compound 7g

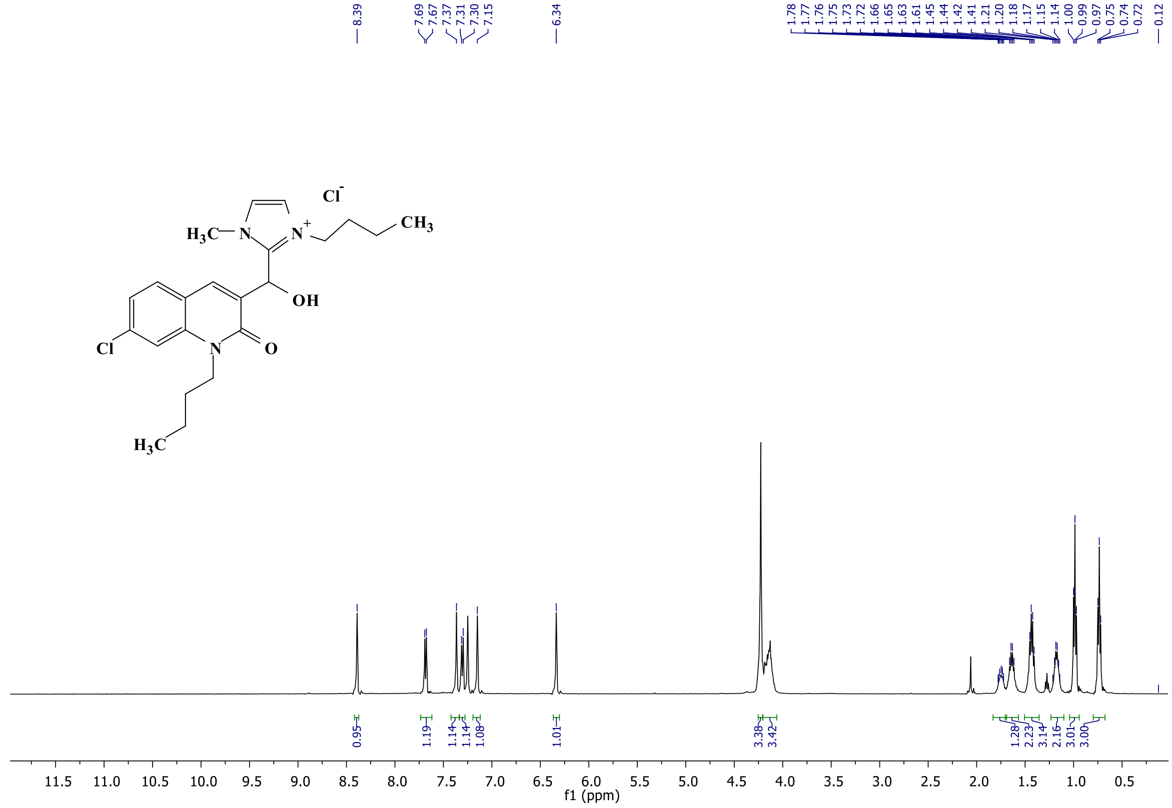

Compound 7h

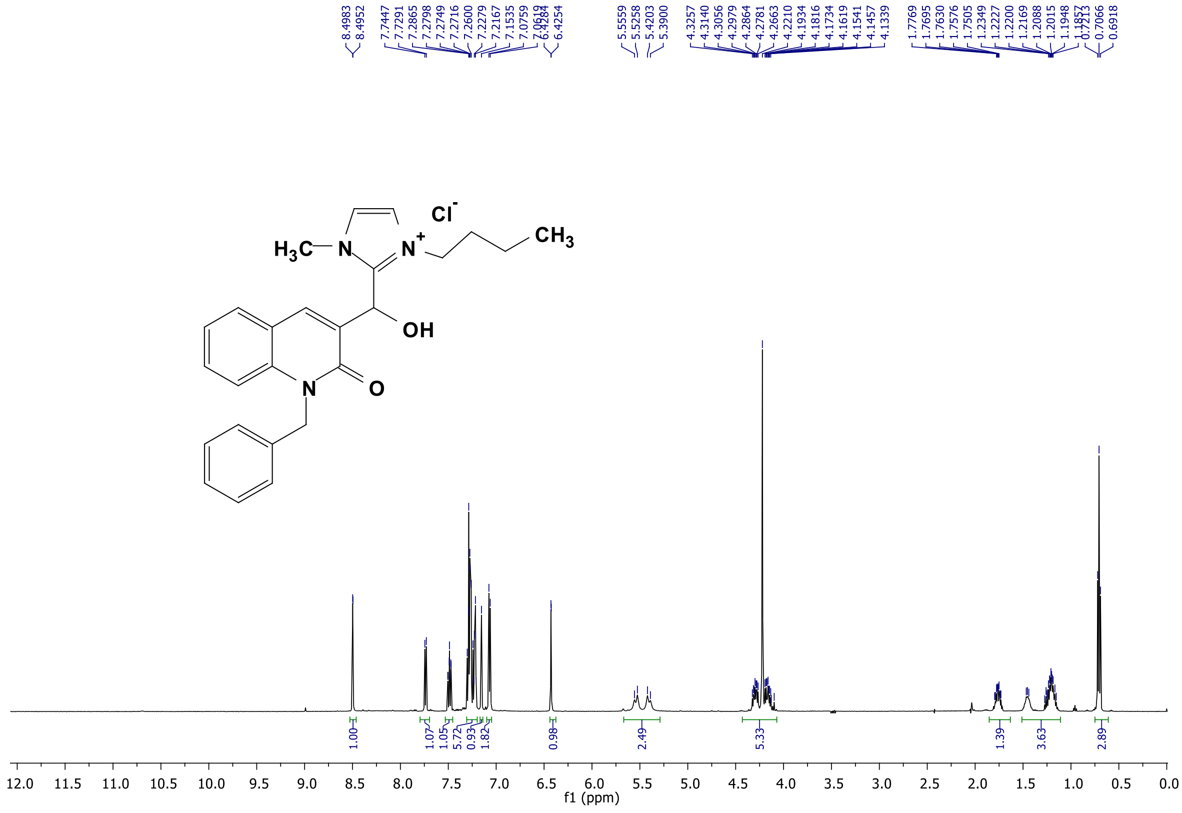

Supplement: Supplementary file 1 [file antibiotics-08-00239-s001.pdf]
